# Large shift of the Pacific Walker Circulation across the Cenozoic

**DOI:** 10.1093/nsr/nwaa101

**Published:** 2020-05-13

**Authors:** Qing Yan, Robert Korty, Zhongshi Zhang, Chris Brierley, Xiangyu Li, Huijun Wang

**Affiliations:** Nansen-Zhu International Research Centre, Institute of Atmospheric Physics, Chinese Academy of Sciences, Beijing 100029, China; Key Laboratory of Meteorological Disaster/Collaborative Innovation Center on Forecast and Evaluation of Meteorological Disasters, Nanjing University of Information Science and Technology, Nanjing 210044, China; Department of Atmospheric Sciences, Texas A&M University, College Station, TX 77843, USA; Department of Atmospheric Science, School of Environmental Studies, China University of Geosciences, Wuhan 430074, China; NORCE Research Climate, Bjerknes Center for Climate Research, Bergen 5007, Norway; Department of Geography, University College London, London WC1E 6BT, UK; Climate Change Research Center, Chinese Academy of Sciences, Beijing 100029, China; Key Laboratory of Meteorological Disaster/Collaborative Innovation Center on Forecast and Evaluation of Meteorological Disasters, Nanjing University of Information Science and Technology, Nanjing 210044, China

**Keywords:** Pacific Walker Circulation, Cenozoic era, paleoclimate modeling

## Abstract

Fluctuations in the Pacific Walker Circulation (PWC), a zonally oriented overturning cell across the tropical Pacific, can cause widespread climatic and biogeochemical perturbations. It remains unknown how the PWC developed during the Cenozoic era, with its substantial changes in greenhouse gases and continental positions. Through a suite of coupled model simulations on tectonic timescales, we demonstrate that the PWC was ∼38° broader and ∼5% more intense during the Early Eocene relative to present. As the climate cooled from the Early Eocene to the Late Miocene, the width of the PWC shrank, accompanied by an increase in intensity that was tied to the enhanced Pacific zonal temperature gradient. However, the locations of the western and eastern branches behave differently from the Early Eocene to the Late Miocene, with the western edge remaining steady with time due to the relatively stable geography of the western tropical Pacific; the eastern edge migrates westward with time as the South American continent moves northwest. A transition occurs in the PWC between the Late Miocene and Late Pliocene, manifested by an eastward shift (both the western and eastern edges migrate eastward by >12°) and weakening (by ∼22%), which we show here is linked with the closure of the tropical seaways. Moreover, our results suggest that rising CO_2_ favors a weaker PWC under the same land-sea configurations, a robust feature across the large spread of Cenozoic climates considered here, supporting a weakening of the PWC in a warmer future.

## INTRODUCTION

The Cenozoic era, spanning from ∼65 Ma to present, experienced substantial changes in greenhouse gases and continental positions, which created a rich diversity of climates [1]. At high latitudes, the planet moved from a hothouse state with ice-free poles to an icehouse state with ice-covered poles during the era. In the tropics, there is evidence that peak sea surface temperatures (SSTs) reached >35°C in the Early Eocene and then exhibited a long-term decline throughout the Cenozoic, with stronger meridional and zonal temperature gradients as the era progressed [2–5]. These changes in SSTs and their gradients modulate large-scale tropical circulations, hence affecting temperature and precipitation across the planet.

One of the most prominent features of the tropics is the Pacific Walker Circulation (PWC), a zonally oriented overturning circulation in the tropical Pacific driven by the east–west SST gradient. The PWC is characterized by ascending motion over the warm western Pacific and descending motion over the cold eastern Pacific today, in tandem with upper-level westerly and surface easterly winds [6,7]. Fluctuations in the PWC could induce global climatic and biogeochemical influences [7]. Understanding the behavior of the PWC could help advance its predictive skill and translate into better forecasting of extreme weather conditions. To improve our knowledge of the PWC, considerable efforts have been made to examine its variations across a broad spectrum of timescales in Earth history [8–12]. There is an incomplete picture of how the PWC might vary on tectonic timescales, owing to paucity of geological evidence.

Here we present a modeled scenario for the Cenozoic evolution of the PWC, using a set of coupled climate model simulations. Deciphering the Cenozoic evolution of the PWC provides insight into the response of the PWC to extremely high greenhouse gases and its global climatic and biogeochemical influences. Additionally, such an investigation helps advance our knowledge on when and how the modern structure of the PWC was established, given the important role of land-sea configurations in shaping it [13–15].

## VARIATION OF THE PWC ACROSS THE CENOZOIC

**Figure 1.**
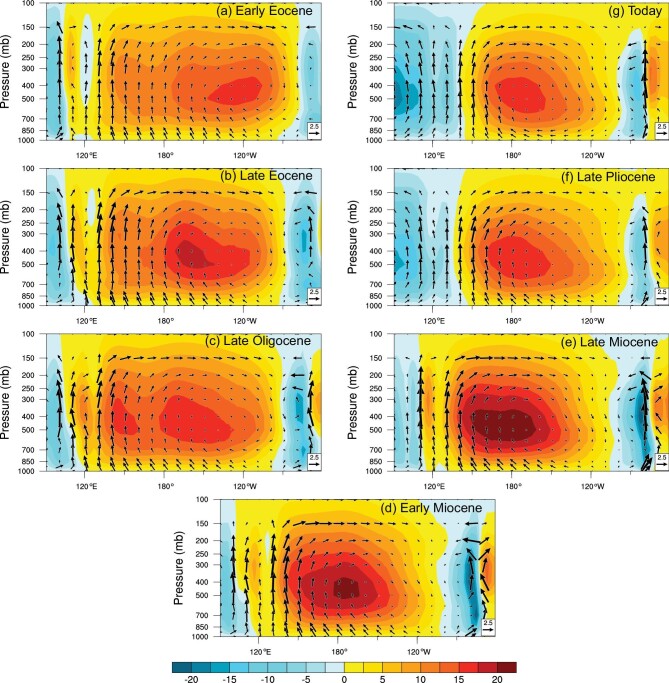
Mean state of the Pacific Walker Circulation during the Cenozoic era. (a) Early Eocene, (b) Late Eocene, (c) Late Oligocene, (d) Early Miocene, (e) Late Miocene, (f) Late Pliocene, and (g) Today. Shadings are the zonal mass streamfunction (10^10^ kg s^−1^) at the equatorial Pacific (5°S–5°N). Positive and negative values represent clockwise and anticlockwise circulations, respectively. Vectors are the composite of pressure velocity (× −50 Pa s^−1^) and zonal divergent wind (m s^−1^). Note that we exclude the ascending motion over the Sundaland (∼90°−120°E) in computing the location and intensity of the PWC (see Methods for details), as it behaves like a local zonal circulation which is broadly separated from the traditional Walker Circulation over tropical Pacific Ocean.

**Figure 2.**
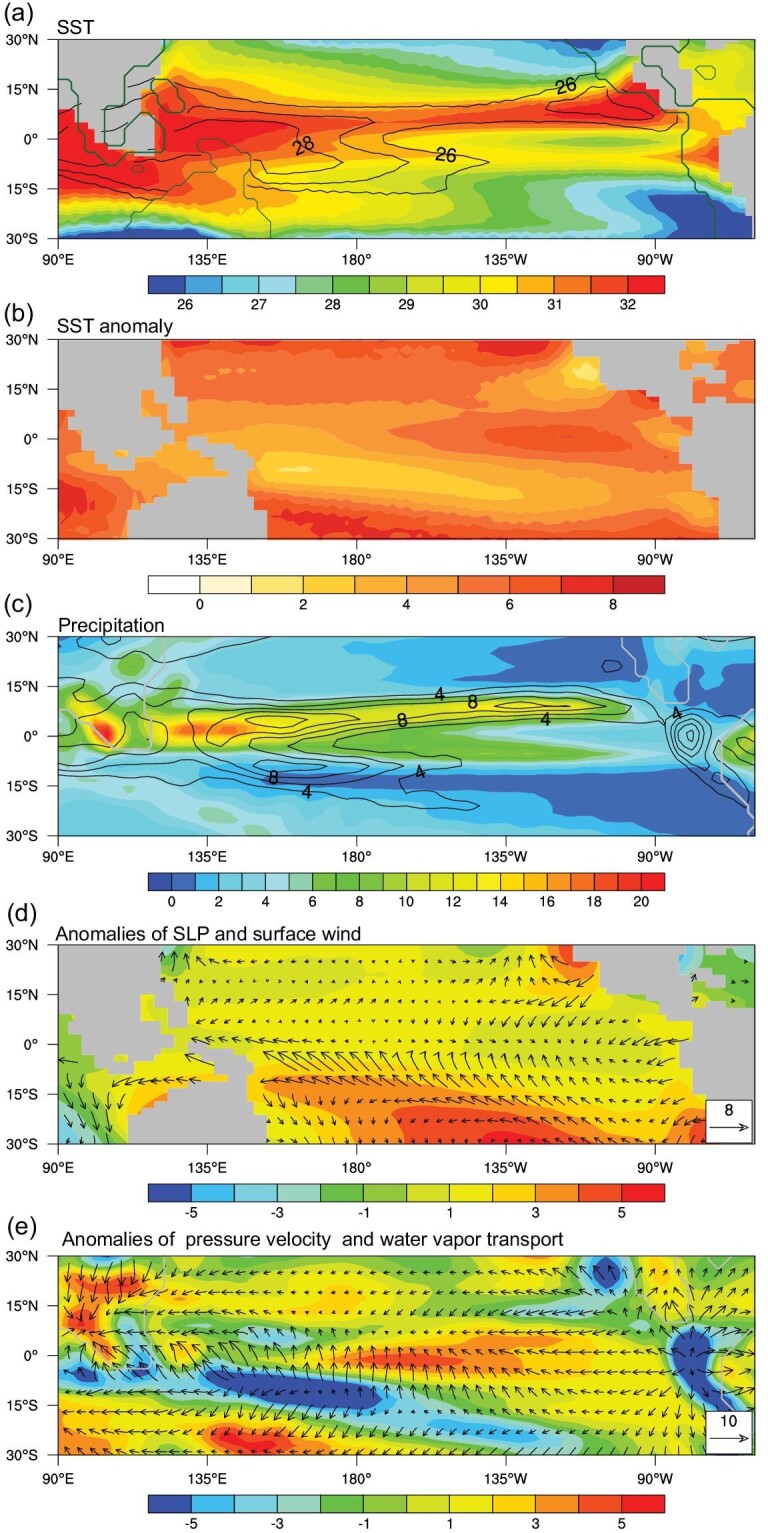
Spatial patterns of environmental variables and their anomalies during the Early Eocene relative to the present. (a) Annual mean sea surface temperature (SST; °C) in the Early Eocene (shadings) and present (contours). The thick dark green lines show the modern land-sea mask in the model. (b) SST anomaly (°C) in the Early Eocene relative to present. (c) Annual precipitation (mm day^−1^) in the Early Eocene (shadings) and present (contours). The thick grey lines show the Early Eocene land-sea mask in the model. (d) Anomalies of annual mean sea-level pressure (SLP; shadings; hPa) and surface wind (vectors; m s^−1^) in the Early Eocene relative to present. Note that the tropical mean of SLP anomalies has been removed. (e) Anomalies of pressure velocity at 500 hPa (shadings; −100 Pa s^−1^) and divergent component of the vertically integrated water vapor transport (vectors; 10^−3 ^kg m^−1^ s^−1^). The thick grey lines show the Early Eocene land-sea mask in the model.

To examine the Cenozoic PWC evolution, we perform a series of coupled climate model simulations across a wide range of Cenozoic climates (see the Data and Methods section), which include the Early Eocene, Late Eocene, Late Oligocene, Early Miocene, Late Miocene, Late Pliocene and pre-industrial. Our results highlight that the intensity and location of the PWC vary substantially during the Cenozoic (Fig. 1). During the Early Eocene (ca. 54–48 Ma), the western edge of the PWC was ∼18° west of its present position, in tandem with a 20° eastward expansion of the eastern edge. This leads to a significant broadening of the PWC by ∼38° (Fig. 1a and Table S1). The broader PWC largely arose from the adjustment of tropical ocean temperatures. During the Early Eocene, when the Pacific was larger in size, the warmest waters (i.e. the ‘warm pool’) were centered more to the northwest than present in our simulations (Fig. 2a and b). Correspondingly, the location of maximum precipitation moved to 120°−150°E compared to ∼150°−180°E today (Fig. 2c), together with a westward shift of ascending motion (Fig. S1). This points to a significant westward expansion of the PWC during the Early Eocene (Fig. 1a and g). The locations of coastal upwelling zones in the eastern tropical Pacific were likely displaced eastward during the Early Eocene relative to present, as was the South American continent (Fig. 2a and Fig. S1). This leads to a corresponding eastward shift of the locations of descending motion and minimum precipitation (Fig. 2c and e and Fig. S1); hence, the eastern edge of the PWC extends further east (Fig. 1a and g). These results suggest a greatly expanded PWC (by ∼38°) in the Early Eocene relative to present (Fig. S2). Notably, a local precipitation center occurred over the west coast of the Sundaland (∼90°−120°E) due to orographic effects (Fig. 2c), closely linked with ascending motion there (Fig. S1). It is unclear if this ascending motion (Fig. 1a) is part of the Pacific component rather than the Indian component of the Walker Circulation, but if included then the PWC in the Early Eocene would have been an additional ∼18° wider.

The intensity of the PWC, defined as the vertically-integrated mass streamfunction averaged across the equatorial Pacific (see the Data and Methods section), was ∼5% stronger in the Early Eocene. There is a large positive sea level pressure anomaly (relative to the tropical mean) over the central south tropical Pacific in the Early Eocene simulation (Fig. 2d), consistent with the oceanic warming pattern (i.e. relatively lower SSTs are associated with higher sea level pressure). This leads to enhanced easterly winds over the western equatorial Pacific (Fig. 2d), accompanied by anomalous convergence of water vapor and ascending motion (Fig. 2e), contributing to the stronger PWC during the Early Eocene (Fig. S2).

**Figure 3.**
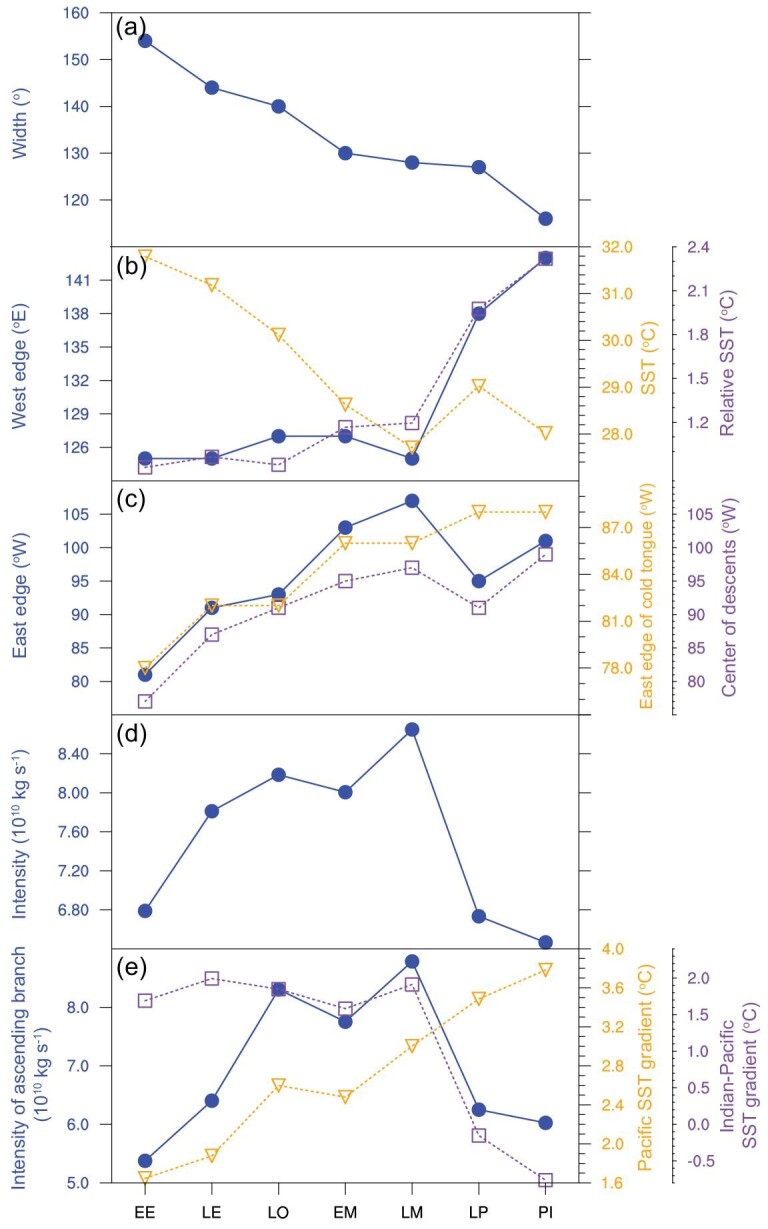
The intensity and location of the Pacific Walker Circulation (PWC) during the Cenozoic era. (a) The width of the PWC (degrees longitude). (b) The location of the western edge of the PWC (blue dots; °E). Orange triangles and purple squares are the SST (°C) and relative SST (°C) averaged over the western equatorial Pacific (5°S–5°N; 120°–180°E), respectively. (c) The locations of the eastern edge of the PWC (°W), the eastern edge of the ‘cold tongue’ (defined as the position of −0.5°C isotherm in relative SST; orange triangles; °W), and the center of descending motion over the eastern tropical Pacific (defined as the location of maximum vertical potential at 200 hPa; purple squares; °W). (d) The intensity of the PWC (10^10^ kg s^−1^). (e) The intensity of the ascending branch of the PWC (10^10^ kg s^−1^). Orange triangles and purple squares are the SST gradient across the equatorial Pacific (defined as the SST difference between 130°–160°E and 150°–120°W; °C) and across the Indian and Pacific Oceans (defined as the SST difference between 60°–90°E and 130°–160°E; °C), respectively. EE, Early Eocene; LE, Late Eocene; LO, Late Oligocene; EM, Early Miocene; LM, Late Miocene; LP, Late Pliocene; PI, pre-industrial.

As the climate cooled from the Early Eocene to the Late Miocene, the width of the PWC reduced, with a long-term increase in intensity (Fig. 3a and d). However, the western and eastern edges of the PWC behave differently. The western edge of the PWC remains relatively constant from the Early Eocene to the Late Miocene (Fig. 3b). Although the tropical Pacific cools with time (Fig. 3b), the western tropical Pacific maintains its warmth relative to the east and is relatively cooler to the west (Fig. S3), as changes in positions of the Asian and Australasian continents are insufficient to drastically alter the tropical oceanographic conditions. Hence, the locations of the maximum precipitation and associated ascending motions are relatively stable with time (Figs S4 and S5), limiting changes in the western edge of the PWC. In contrast, the South American continent drifts northwestward from the Early Eocene to the Late Miocene (Fig. S3), causing the position of the cold water over the eastern tropical Pacific to shift westward with the land boundary (Fig. 3c). This contributes to a westward migration of descending motion that is clearly manifested in the velocity potential at 200 hPa (Fig. 3c), indicating a westward shift in the eastern edge of the PWC. The intensity of the PWC exhibits an upward trend from the Early Eocene to the Late Miocene (Fig. 3d), dominated by a strengthening of the ascending branch (Fig. 3e). The intensification of the PWC is largely attributed to the enhanced zonal SST gradient over the equatorial Pacific from the Early Eocene to the Late Miocene in our simulations (Fig. 3e).

## THE LATE MIOCENE−LATE PLIOCENE TRANSITION

An obvious shift in the PWC during the Cenozoic era occurred at the transition from the Late Miocene (ca. 11−6 Ma) to the Late Pliocene (ca. 3 Ma; Fig. 3). The western edge of the PWC jumped eastward by ∼13° (caused by the warming of the western equatorial Pacific; Fig. 3b), but was compensated by a coeval 12° expansion of the eastern edge (Fig. 3c), leading to limited change in the width of the PWC. This shift happened in tandem with

a significant weakening of the PWC (by ∼22%). The weaker PWC is linked with the reduced zonal SST gradient and even a reversal of its sign between the Indian Ocean and the western equatorial Pacific (Fig. 3d and e), which overwhelms the role of increased SST gradient across the equatorial Pacific, as seen in the geological evidence [2].

What caused this shift and weakening during the Miocene−Pliocene transition? Two key drivers of climate change on geological timescales are changes in CO_2_ concentrations and the movements of tectonic plates. Given the similar CO_2_ concentrations during the Late Miocene and Pliocene (350 vs. 405 ppmv in our simulations), the large shift of PWC would appear to have been driven by tectonics, among which the restriction of the Indonesian seaway and the closure of the Panama seaway stand out.

**Figure 4.**
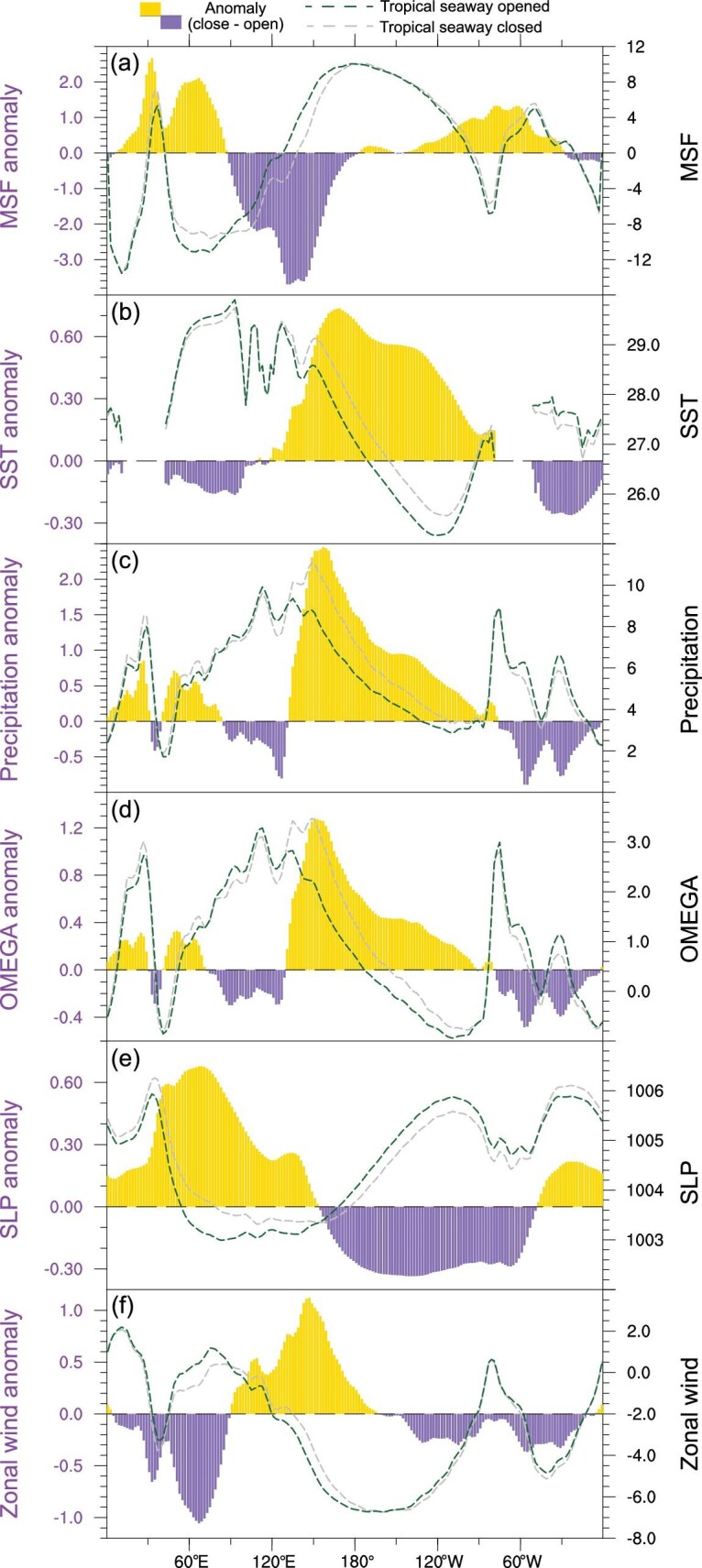
Meridional mean of environmental conditions and their differences caused by the tropical seaway closures. (a) Vertical average of mass stream function (MSF; 10^10^ kg s^−1^). (b) Sea surface temperature (SST; °C). (c) Precipitation (mm day^−1^). (d) Pressure velocity at 500 hPa (OMEGA; × −50 Pa s^−1^). (e) Sea level pressure (SLP; hPa). (f) Surface zonal wind (m s^−1^). The color bars show the differences caused by the tropical seaway closures, whereas the contours show the climatological mean (right axis) in the experiments with the tropical seaway closed (gray dash lines) and opened (darkgreen dash lines).

To test our hypothesis, we design two sensitivity experiments to isolate the climatic effect of the tropical seaway closures (see the Data and Methods section). In one experiment, the Indonesian seaway is broadened and the Panama seaway is opened. In the other experiment, we just open the Panama seaway with the Indonesian seaway closed. The results suggest that the closure of both tropical seaways led to a warming over the equatorial Pacific but cooling over the Indian and Atlantic Oceans (Fig. 4b). These changes in SSTs resulted in increased precipitation (anomalous ascending motion) to the east of ∼125°E and decreased precipitation (anomalous descending motion) to the west (Fig. 4c and d), suggesting an eastward shift of western edge over the western tropical Pacific that is clearly manifested in the variation of the mass stream function (Fig. 4a). Similarly, an eastward migration of eastern edge is also simulated over the eastern tropical Pacific. These results indicate a systematic eastward shift of the PWC due to the closure of tropical seaways (Fig. S6). Moreover, the ‘Indian Ocean-cooling and Pacific-warming’ pattern results in anomalous sea level pressure gradient between Indian Ocean and western tropical Pacific (Fig. 4e). This leads to a westerly anomaly along the western equatorial Pacific (Fig. 4f), contributing largely to the reduced intensity of the PWC.

The eastward shift and weakening of the PWC seen in the tropical seaway closure experiments match well with the PWC changes during the Miocene−Pliocene transition (Fig. 3 and Fig. S7). The magnitude of the changes is underestimated in the sensitivity experiments, which may be partially linked with the relatively smaller depth and width of tropical seaways used in it. Nonetheless, our results highlight the dominant role of tropical seaway closures in driving the PWC shift during the Miocene–Pliocene transition. Furthermore, the restriction of the Indonesian seaway plays a more important role in the eastward shift of the ascending branch of the PWC (Fig. S8), via reducing the Indonesia Throughflow [16]. In contrast, the closure of the Panama seaway has a larger effect on the eastward migration of the descending branch (Fig. S8), by preventing the throughflows between the Atlantic and Pacific. Although changes in land distribution/elevation over the Maritime Continent have been shown to be important for Walker Circulation, its effect is largely concentrated over the Indian Ocean rather than the Pacific [15,17]. The development and expansion of the Antarctic ice sheet has a substantial influence on local/remote climates [18], but an additional sensitivity experiment indicates it has only a limited effect on the structure and intensity of the PWC (Fig. S9), as the response of deep tropics is much smaller (<1°C), with a zonal symmetric pattern.

## CONCLUSION

Here we present a modeled scenario for the Cenozoic evolution of the PWC. The width of the PWC narrowed significantly throughout the Cenozoic era, accompanied, until the Pliocene, by a strengthening. The narrowing largely arose from the eastern edge of the PWC migrating westward, driven by the northwestward movement of the South American continent. There was an eastward shift (by ∼12°) of the whole PWC sometime between the Late Miocene and Late Pliocene, for which the closure of the Indonesian and Panama seaways was largely responsible. The intensity of the PWC is strongly related to the equatorial Pacific zonal temperature gradient from the Early Eocene to the Late Miocene, whereas it reduces by ∼22% during the Miocene−Pliocene transition arising from the reduced SST contrast between Indian Ocean and western Pacific.

Notably, the intensity of the PWC was stronger during the Early Eocene than present, the closest geologic analogue for future climate under unmitigated emission trajectories [19]. This seems opposite to the projected weakening of the PWC at the end of the 21st century [20,21] or under idealized warming scenarios [22]. Further sensitivity experiments that separate the influences of CO_2_ and land-sea configurations illustrate that rising CO_2_ alone leads to a weaker PWC, a robust feature across the large spread of Cenozoic climates considered here (Fig. S10) and therefore in a warmer future. Our results also highlight that, at least on tectonic timescales, the location of the PWC is largely controlled by plate movements, with CO_2_ concentrations playing a secondary role impacting solely the intensity (Fig. S10).

Although there is increasing evidence for SST variations over the central-east Pacific in deep times [23,24], there is a lack of SST records over the equatorial western Pacific, hampering the effort to reconstruct the zonal SST gradient across the Paleogene. Thus, the modeled evolution of PWC intensity from the Early Eocene to Late Miocene is difficult to constrain directly by geologic evidence, but a shrinkage during the Cenozoic era had been proposed [25]. Furthermore, the tropical seaways are generally considered to be closed during the Pliocene [26–28], leading to the cooling over the Indian Ocean and warming over the western tropical Pacific seen in geological evidence [26,29–31]. The interbasin warming contrast favors a weakening of the PWC due to the expected anomalous west-to-east sea level pressure gradient and hence the westerly over western tropical Pacific [32], confirmed by our simulations. In addition to the PWC, both the Atlantic and Indian cells also experienced substantial changes across the Cenozoic (Fig. S11). Particularly, there are only four loops of zonal circulations across the tropics from the Early Eocene to Late Oligocene, in contrast to the six loops observed in the modern climate. This is attributed to the wider Indian cell and near-disappearance of the loop over North Africa (∼40°E). However, additional proxies for SST over the deep tropics are necessary to test the modeled evolution of the PWC during the Cenozoic. It should be noted that here we focus on the change in the mean state of the PWC on tectonic timescales, but there are still clearly interannual variations of the PWC in each period, i.e. the edge of the PWC shifts substantially with the El Niño-Southern Oscillation.

Although there are uncertainties to be considered, our study provides a testable relationship between tectonic/CO_2_-induced climate change and the behavior of the PWC. The substantial changes in the PWC simulated here serve as a potential factor responsible for the reconstructed hydrological changes across the globe during the Cenozoic era. Moreover, a comprehensive understanding of the controls of the PWC could help advance its predictive skill and translate into better forecasting of extreme weather conditions.

## DATA AND METHODS

### Climate model

We use the low-resolution version of the Norwegian Earth System Model (NorESM-L) [33], a fully coupled climate model designed for paleoclimate simulations [34], to examine climate change during the Cenozoic era. The atmospheric component of the NorESM-L is the Community Atmospheric Model version 4, which has a horizontal resolution of  ∼3.75° × 3.75° with 26 vertical levels. The Miami Isopycnic Coordinate Ocean Model serves as the ocean component, with a nominal 3° horizontal resolution and 32 vertical levels. The land and sea ice component is Community Land Model version 4 and Los Alamos Sea Ice Model version 4, respectively. The NorESM-L has been widely used in modeling past climates in Earth history on various timescales and proven skillful in reproducing the majority of reconstructed climatic features [34–36].

### Experimental design

We carry out a suite of deep-time experiments with the NorESM-L to simulate a wide range of Cenozoic climates. These experiments target the climate of the Early Eocene, Late Eocene, Late Oligocene, Early Miocene, Late Miocene, Late Pliocene and today (Table S2). The land-sea configurations from the Early Eocene to the Late Miocene are derived from the reconstructed paleogeographic maps [37], whereas the Late Pliocene topography is based on the Pliocene Research, Interpretation and Synoptic Mapping (PRISM) project [38]. Based on a synthesis of atmospheric CO_2_ concentrations [39], we set atmospheric CO_2_ concentrations to 1120 ppmv for the Early Eocene, 1050 ppmv for the Late Eocene, 700 ppmv for the Late Oligocene, 420 ppmv for the Early Miocene, 350 ppmv for the Late Miocene and 405 ppmv for the Late Pliocene. For the land cover, we use the same idealized vegetation for each of these deep-time simulations owing to the scarcity of proxies for vegetation during most of the Cenozoic, except for the Late Pliocene for which we adopt the PRISM3D reconstructions [38]. Additionally, we rerun these deep-time experiments but with the CO_2_ concentration fixed at 560 ppmv to isolate the effect of continental configurations and CO_2_ concentration. Each experiment is integrated for 2200 model years, and we analyze the outputs of the last 200 years.

To test the climatic effects of the closure of tropical seaways, we perform another two sensitivity experiments. In one sensitivity experiment, we open both the Indonesian and Panama seaways, leaving the other boundary conditions unchanged from the Late Pliocene run. In the other sensitivity experiment, only the Panama seaway is opened. The modifications of tropical seaways in these sensitivity experiments are designed to roughly represent Pliocene palaeoceanographic conditions, as geological evidence suggests that the tropical seaways of Indonesia and Panama were likely closed during the Pliocene [26–28]. Each experiment is integrated for 1 500 model years, and we analyze the output of the last 200 years. More details concerning the experimental set-up can be found in Ref. [16].

Additionally, we performed a pair of experiments for the Early Miocene (EM and EM_ice) to study the effect of Antarctic ice sheet (Table S2). In the experiment EM, we set CO_2_ concentration to 420 ppmv and use idealized vegetation, in place of an Antarctic ice sheet. In the experiment EM_ice, a modern Antarctic ice sheet is prescribed in the model, with the other settings identical to the experiment EM [18]. The experiment EM_ice is initialized from the experiment EM and is integrated for 1200 model years, and we analyze the output of the last 100 years.

### Pacific Walker Circulation

As the PWC is a thermally driven equatorial Pacific zonal circulation, it is directly measured by the zonal mass streamfunction ($\psi $) [40,41]:

(1) \begin{equation*} \psi {\rm{ = }}\frac{{{\rm{a}}\Delta \varphi }}{{\rm{g}}}\int_{0}^{{\rm{p}}}{{{u_{\rm{D}}}{\rm{d}}p,}} \end{equation*}

where ${u_{\rm{D}}}$ is the divergent component of the zonal wind that is closely connected to divergence/convergence and hence vertical motion, $\Delta \varphi $ is the width of the band 5°S–5°N, *p* is the pressure, a is the radius of Earth and g is the gravity acceleration. In comparison with the other PWC indices (e.g. east–west sea level pressure/surface wind gradient [20,42]), the mass streamfunction is a direct measure of the two-dimensional structure of the PWC, as the PWC can be mathematically represented by a line integral of tangential wind speed along a closed circle over the equatorial Pacific vertical sector [43]. Another advantage is that the calculation of mass streamfunction is not affected by changes in land-sea configurations, but may influence the choice of key regions when using sea level pressure/surface wind gradient.

In general, the western and eastern edges of the PWC are measured as the zero line of the vertically integrated (from surface to the top of the model) zonal mass streamfunction on the west and east side of the international date line, respectively. However, the ascending motion over the Sundaland (∼90°−120°E) from the Early Eocene and Late Miocene behaves as a local zonal circulation and roughly separates from the traditional Walker Circulation over tropical Pacific Ocean (e.g. Fig. 1a). Here, we define the western edge as the longitude of the minimum zonal mass streamfunction between ∼120°E and the international date line to exclude the influence of ascending motion over the Sundaland. The intensity of the PWC is defined as vertically integrated zonal mass streamfunction averaged across the equatorial Pacific (i.e. from the western to the eastern edge of the PWC). For the intensity of western edge (i.e. ascending branch), it is defined as vertically integrated zonal mass streamfunction averaged over a 40° band starting from the western edge of the PWC.

## Supplementary Material

nwaa101_Supplemental_File
