# Supplementary material for: Large shift of the Pacific Walker Circulation across the Cenozoic
Source: Natl Sci Rev. 2020 May 13;8(5):nwaa101. doi: 10.1093/nsr/nwaa101 (PMC8288383; doi:10.1093/nsr/nwaa101)
Supplement: nwaa101_Supplemental_File [file nwaa101_supplemental_file.pdf]

Supporting information for

Large shift of the Pacific Walker Circulation across the Cenozoic

Qing Yan*, Robert Korty, Zhongshi Zhang*, Chris Brierley, Xiangyu Li, and Huijun Wang

(yanqing@mail.iap.ac.cn; zhongshi.zhang@cug.edu.cn)

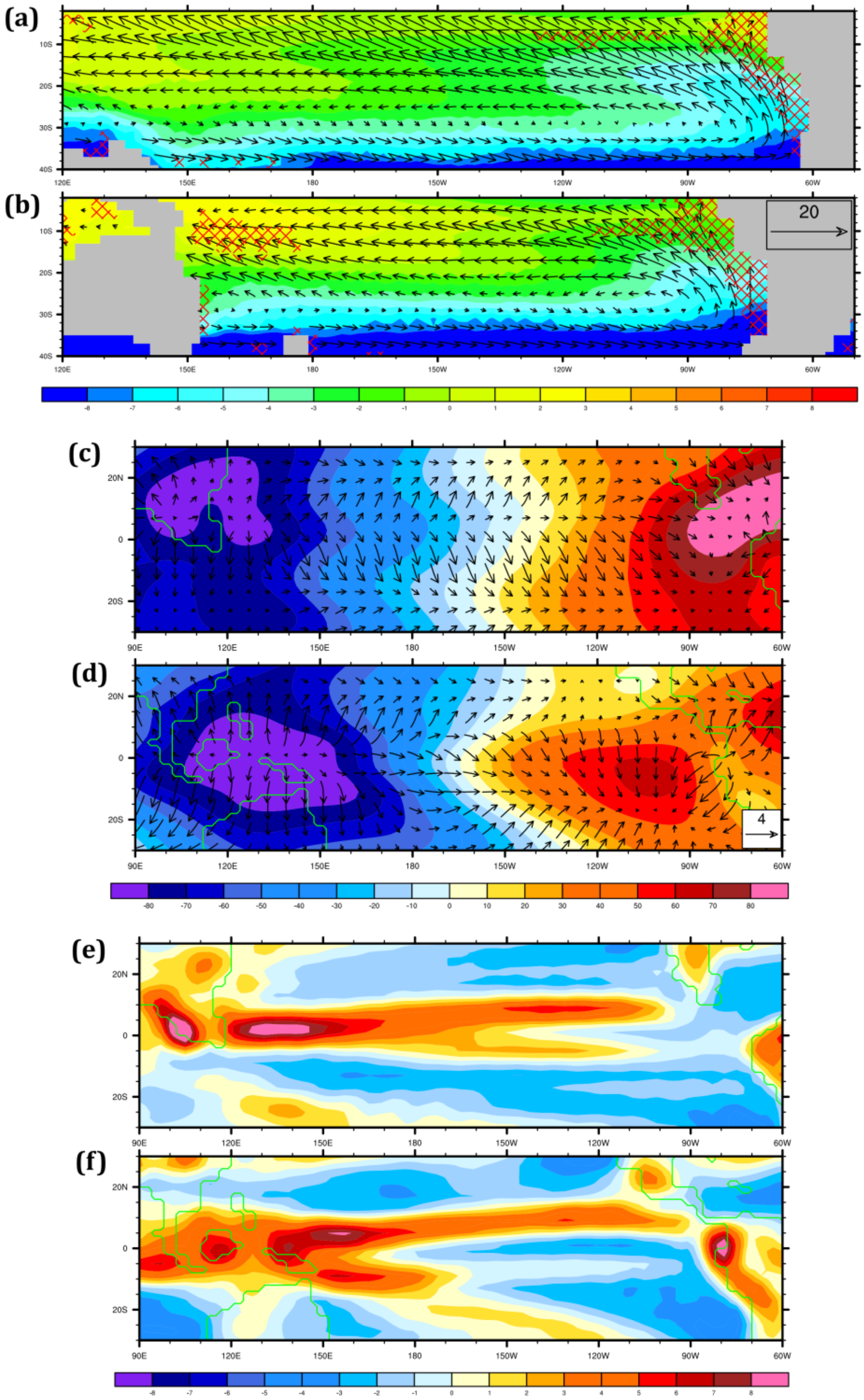

Figure S1. (a, b) Distribution of relative SST (shadings; ºC), surface wind (m s^{-1}), and upwelling (hatches) in the (a) Early Eocene and (b) today. (c, d) Distribution of velocity potential at 200 hPa (shadings; $\times 10^5$m^2 s^{-1}) and divergent wind (m s^{-1}) in the (c) Early Eocene and (d) today. (e, f) Distribution of pressure velocity at 200–850 hPa ($\times$–100 Pa s^{-1}) in the (e) Early Eocene and (f) today. Note that the accurate locations of coastal upwellings may be biased somehow due to the coarse model resolution, but the westward migration from the Early Eocene to present should be largely held.

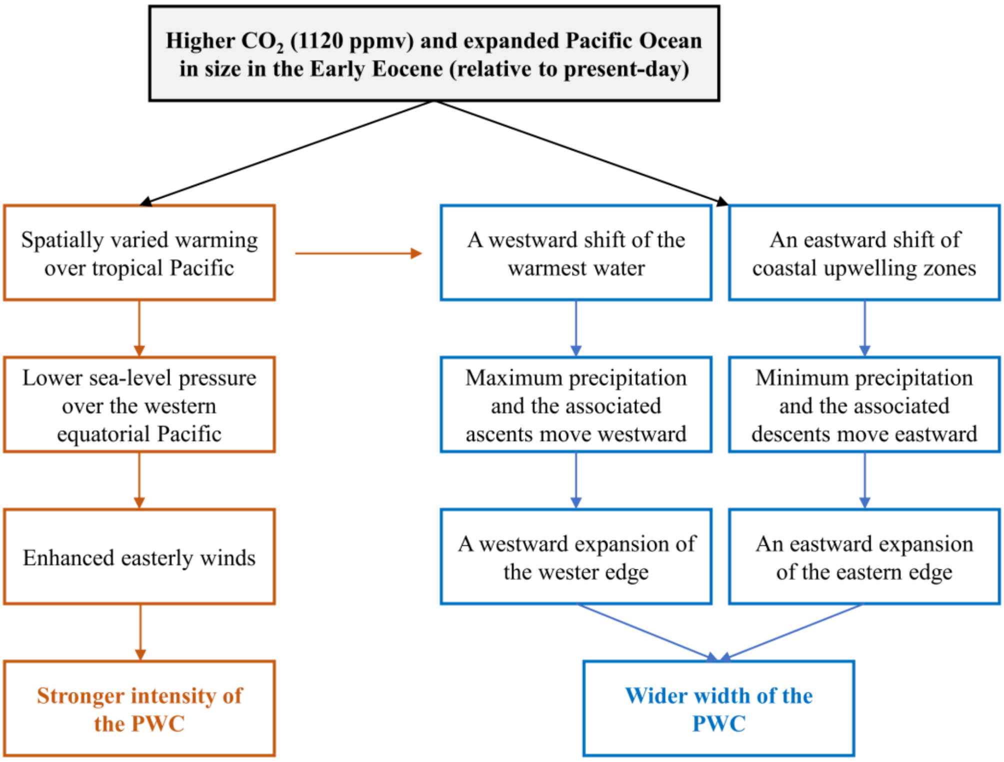

Figure S2. Schematic diagram illustrating the main processes linked with the variation of the PWC during the Early Eocene relative to present-day.

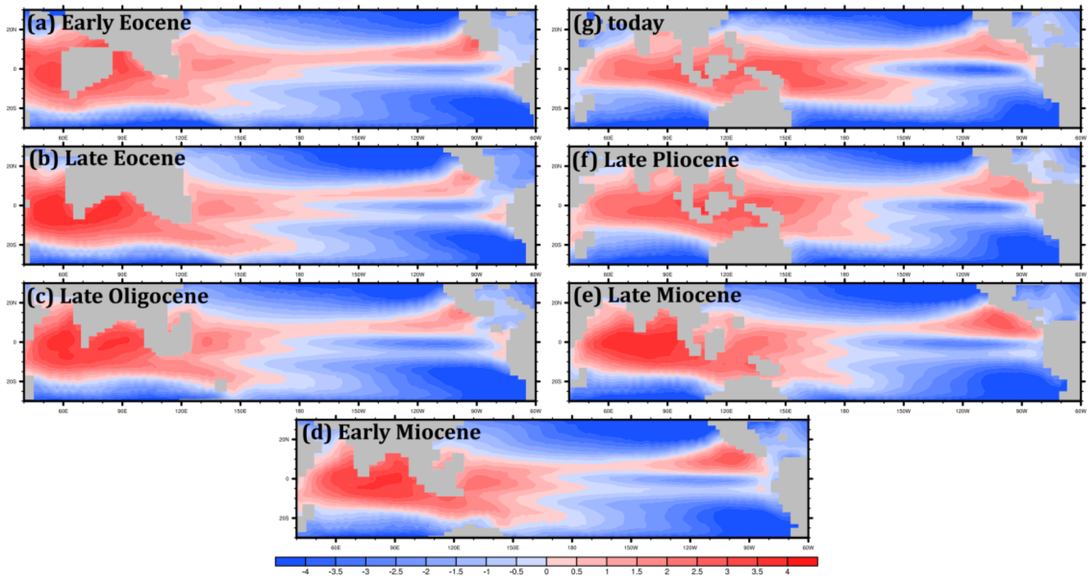

Figure S3. Distribution of relative SST (ºC) in the (a) Early Eocene, (b) Late Eocene, (c) Late Oligocene, (d) Early Miocene, (e) Late Miocene, (f) Late Pliocene, and (g) today.

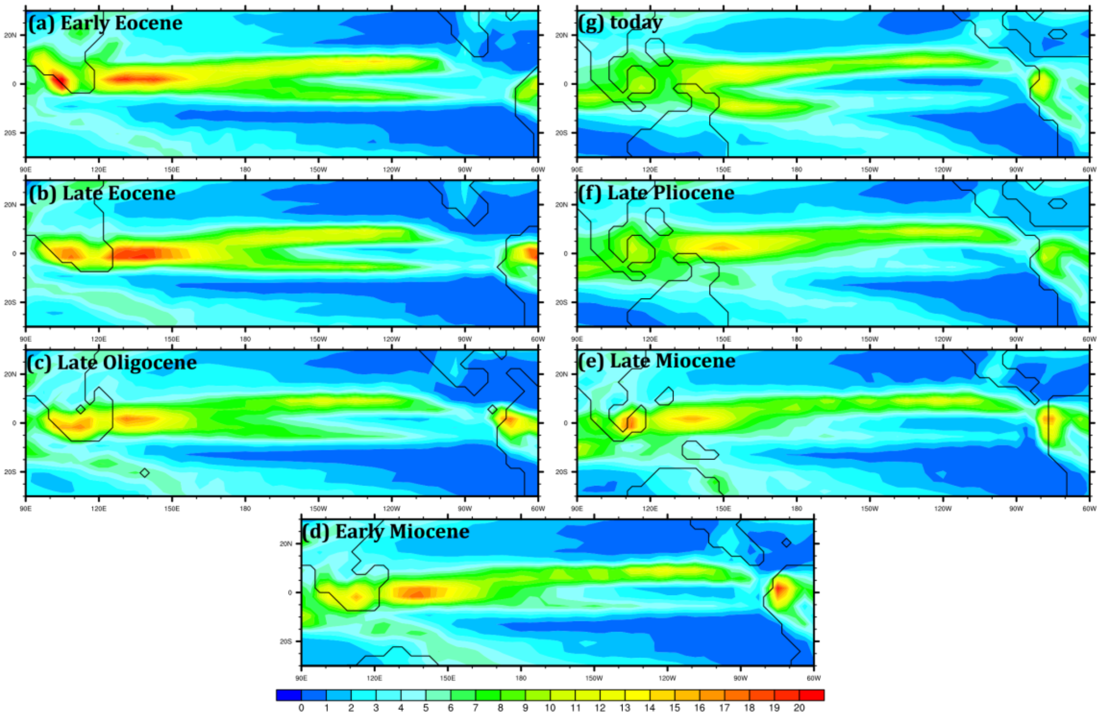

Figure S4. Distribution of precipitation (mm/day) in the (a) Early Eocene, (b) Late Eocene, (c) Late Oligocene, (d) Early Miocene, (e) Late Miocene, (f) Late Pliocene, and (g) today.

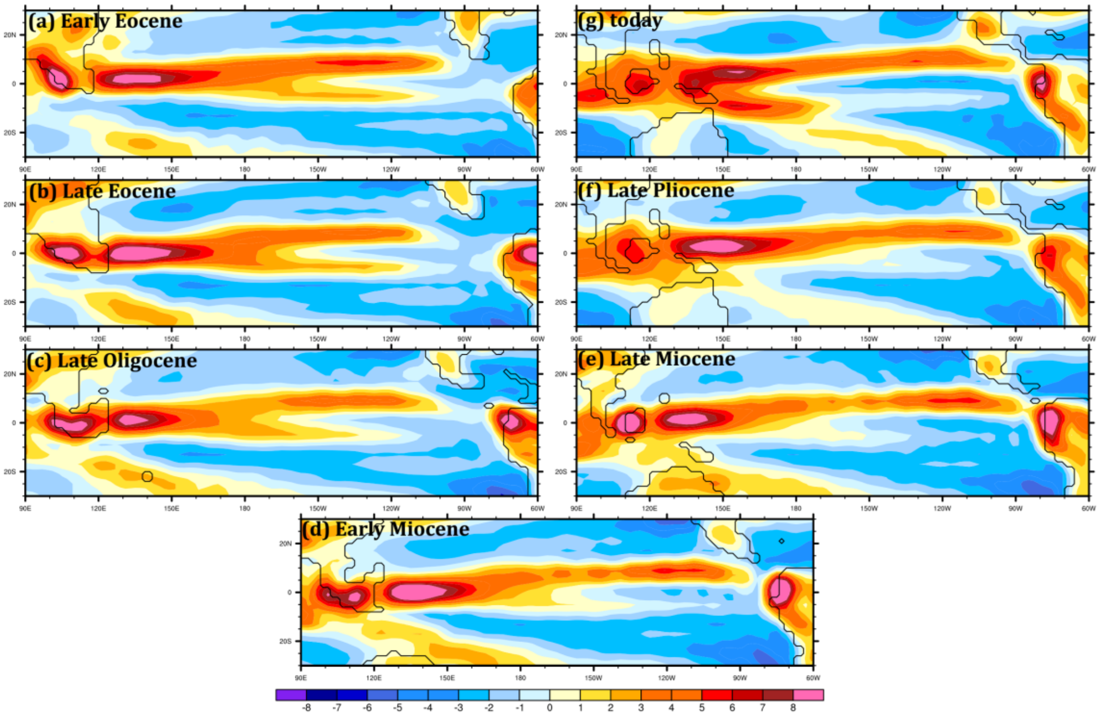

Figure S5. Distribution of pressure velocity at 200–850 hPa (×–100 Pa s^{-1}) in the (a) Early Eocene, (b) Late Eocene, (c) Late Oligocene, (d) Early Miocene, (e) Late Miocene, (f) Late Pliocene, and (g) today.

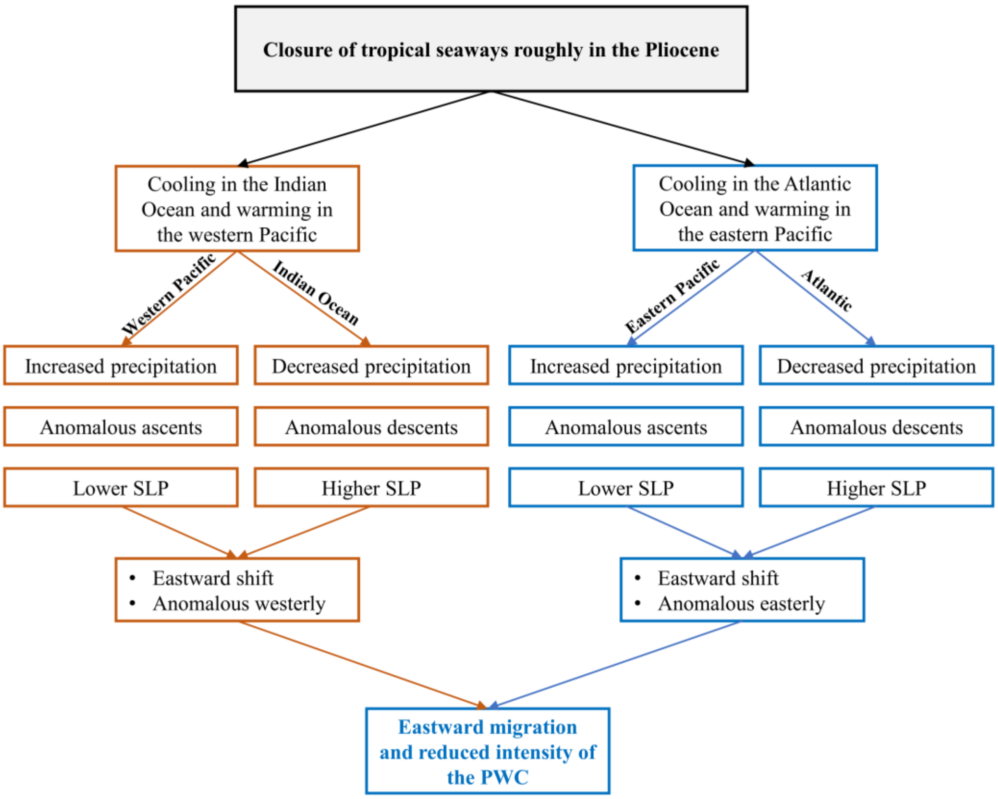

Figure S6. Schematic diagram illustrating the main processes linked with the variation of the PWC due to the closure of tropical seaways.

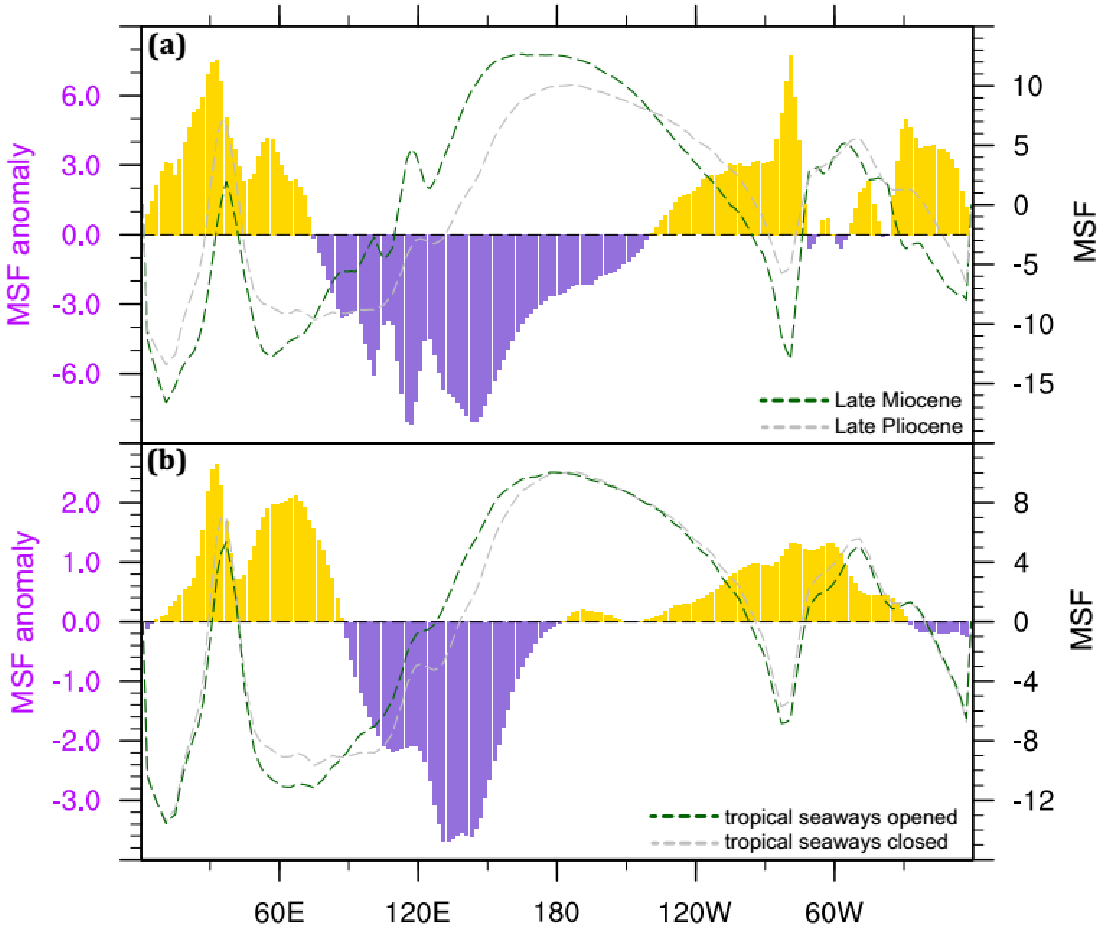

Figure S7. Mass stream function (right axis) and its difference (left axis) (a) during the Mio-Pliocene transition (i.e., Late Pliocene minus Late Miocene) and (b) caused by the closure of tropical seaways. (a) The contours show the climatological mean in the Late Pliocene (gray dash lines) and the Late Miocene (darkgreen dash lines). (b) The contours show the climatological mean in the experiments with the tropical seaway closed (gray dash lines) and opened (darkgreen dash lines). Units: 10^{10} kg s^{-1}.

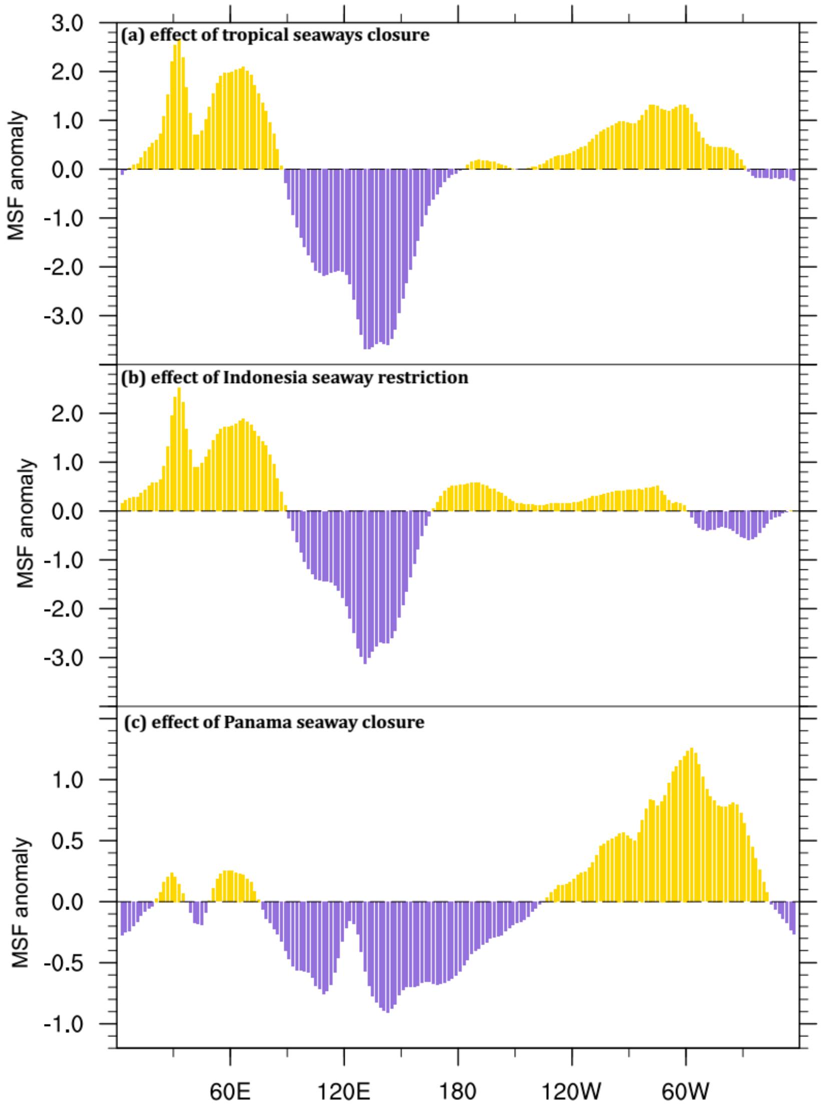

Figure S8. Differences in vertical average of mass stream function (10^{10} kg s^{-1}) caused by the closure of (a) tropical seaway, (b) only the Indonesia seaway, and (c) only the Panama seaway.

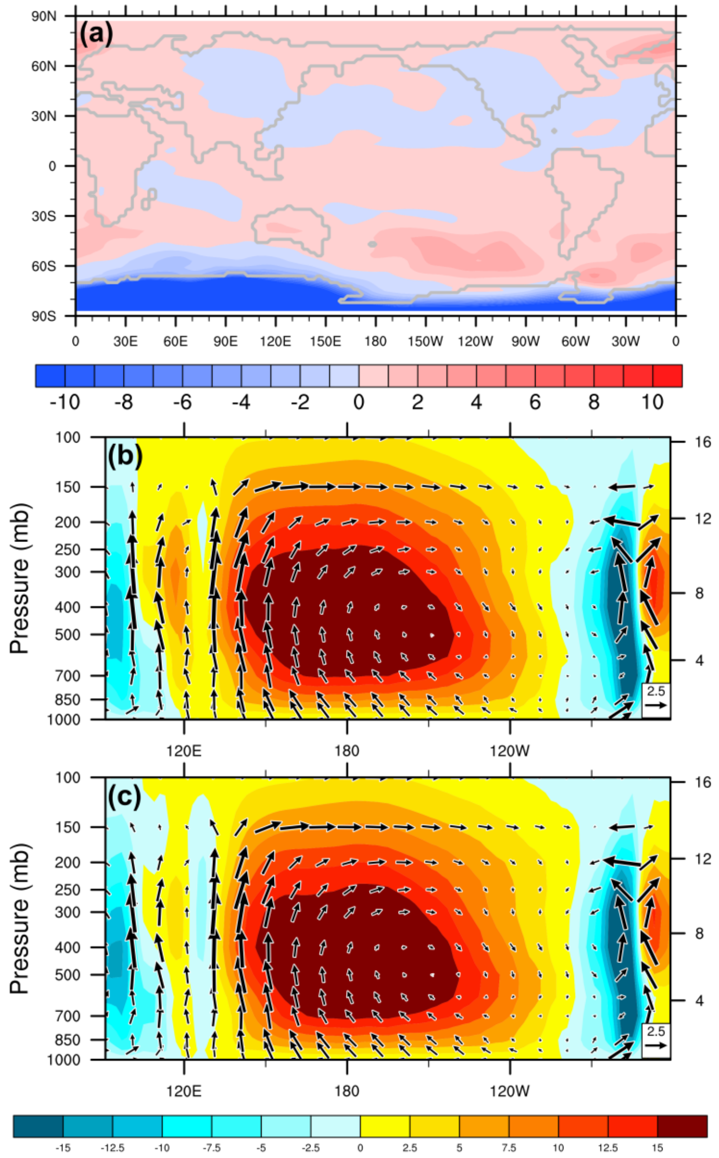

Figure S9. (a) Temperature anomaly induced by the formation of the Antarctic ice sheet based on the Late Miocene boundary conditions. (b, c) Mean state of the Pacific walker circulation during the Late Miocene with (b) and without (c) the Antarctic ice sheet. The mean intensity of the PWC in (b) and (c) is 8.36 and 8.37×10^{10} kg s^{-1}, respectively.

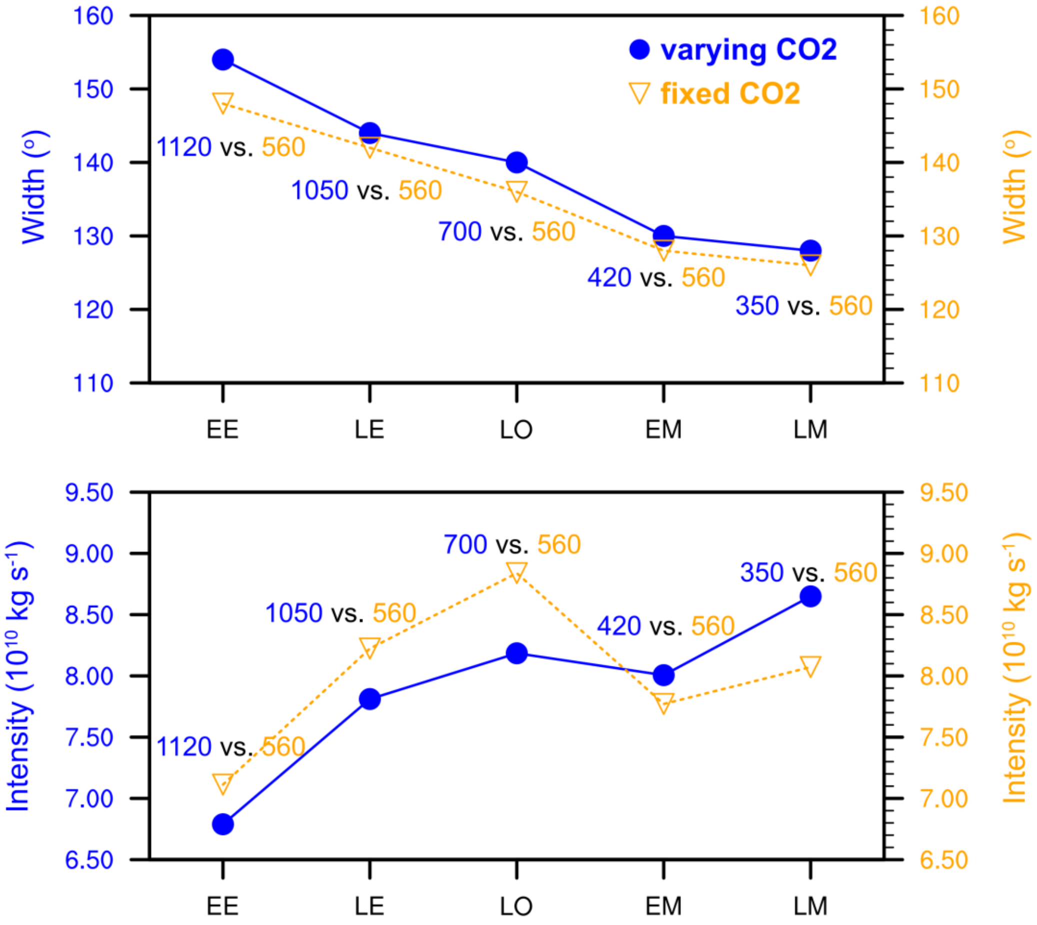

Figure S10. The width (a; degrees longitude) and intensity of the PWC (b; 10^{10} kg s^{-1}) during the Cenozoic era under different CO_2 levels. The blue dots show the PWC intensity in different Cenozoic climates with varying CO_2 concentrations, while the orange triangles show the intensity in different Cenozoic climates with the fixed CO_2 concentrations (560 ppmv). EE: Early Eocene, LE: Late Eocene, LO: Late Oligocene, EM: Early Miocene, LM: Late Miocene.

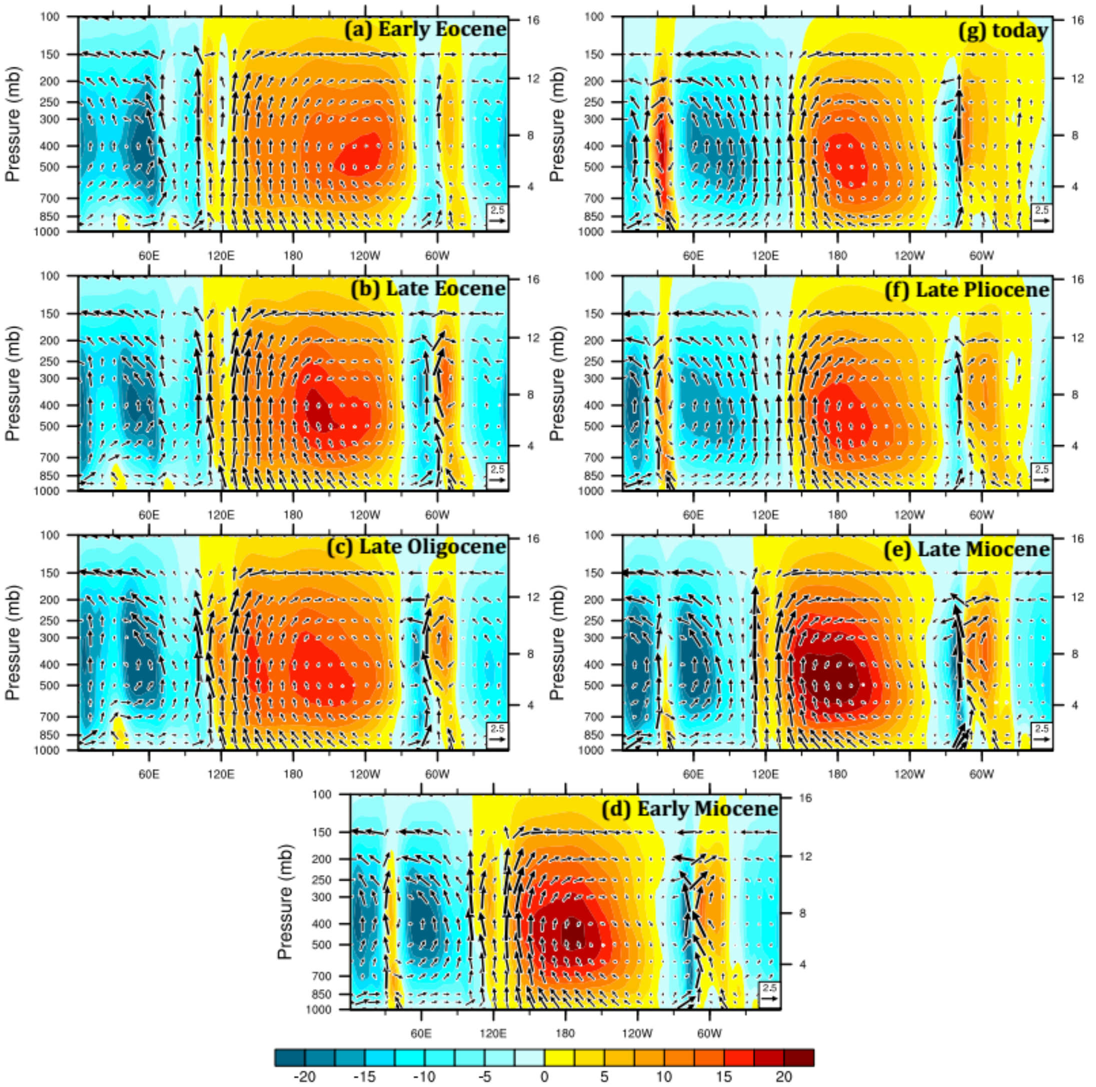

Figure S11. Mean state of the walker circulation during the Cenozoic era. (a) Early Eocene, (b) Late Eocene, (c) Late Oligocene, (d) Early Miocene, (e) Late Miocene, (f) Late Pliocene, and (g) today. Shadings are the zonal mass streamfunction (10^{10} kg s^{-1}) at the equatorial Pacific (5ºS–5ºN). Positive and negative values represent clockwise and anticlockwise circulations, respectively. Vectors are the composite of pressure velocity (×–50 Pa s^{-1}) and zonal divergent wind (m s^{-1}).

Table S1. Metrics for the Pacific walker circulation during the Cenozoic era.

EXP.	West edge (ºE)	East edge (ºW)	Width (º)	Intensity (10^{10} kg s^{-1})
EE_1120	125	81	154	6.8
LE_1050	125	91	144	7.8
LO_700	127	93	140	8.2
EM_420	127	103	130	8.0
LM_350	125	107	128	8.6
LP_405	138	95	127	6.7
PI_280	143	101	116	6.5

Table S2. Experimental design for the Cenozoic climates

EXP	CO_2 (ppmv)	Geography	Vegetation
a, varying CO_2			
EE_1120	1120	Early Eocene	Idealized[*]
LE_1050	1050	Late Eocene	Idealized
LO_700	700	Late Oligocene	Idealized
EM_420	420	Early Miocene	Idealized
LM_350	350	Late Miocene	Idealized
LP_405	405	Late Pliocene	Late Pliocene
PI_280	280	modern	modern
b, fixed CO_2			
EE_560	560	Early Eocene	Idealized
LE_560	560	Late Eocene	Idealized
LO_560	560	Late Oligocene	Idealized
EM_560	560	Early Miocene	Idealized
LM_560	560	Late Miocene	Idealized
c, effect of tropical seaway closure			
LP_close[#]	405	modern	Late Pliocene
LP_openP	405	modern with the Panama seaway opened	Late Pliocene
LP_openIP	405	modern with the Indonesia and Panama seaways opened	Late Pliocene
d, effect of Antarctic ice sheet			
EM[%]	420	Early Miocene	Idealized without Antarctic ice sheet
EM_ice	420	Early Miocene	Idealized with Antarctic ice sheet

[*]Forest is prescribed between 30ºS and 30ºN, and shrub and grass outside this latitude band without polar ice sheets.

[#]LP_close and LP_405 are the same experiment.

[%]EM and EM_420 are the same experiment.
